# Prevalence and Patterns of Tobacco Use in Bangladesh from 2009 to 2012: Evidence from International Tobacco Control (ITC) Study

**DOI:** 10.1371/journal.pone.0141135

**Published:** 2015-11-11

**Authors:** Nigar Nargis, Mary E. Thompson, Geoffrey T. Fong, Pete Driezen, A. K. M. Ghulam Hussain, Ummul H. Ruthbah, Anne C. K. Quah, Abu S. Abdullah

**Affiliations:** 1 American Cancer Society, Washington DC, United States of America; 2 University of Dhaka, Dhaka, Bangladesh; 3 University of Waterloo, Waterloo, Ontario, Canada; 4 Boston University School of Medicine, Boston, Massachusetts, United States of America; 5 Global Health Program, Duke Kunshan University, Jiangsu, China; BRAC, BANGLADESH

## Abstract

**Background:**

Smoking and passive smoking are collectively the biggest preventable cause of death in Bangladesh, with major public health burden of morbidity, disability, mortality and community costs. The available studies of tobacco use in Bangladesh, however, do not necessarily employ nationally representative samples needed to monitor the problem at a national scale. This paper examines the prevalence and patterns of tobacco use among adults in Bangladesh and the changes over time using large nationally representative comparable surveys.

**Methods:**

Using data from two enumerations of the International Tobacco Control (ITC) Bangladesh Project conducted in 2009 and 2012, prevalence estimates are obtained for all tobacco products by socio-economic determinants and sample types of over 90,000 individuals drawn from over 30,000 households. Household level sample weights are used to obtain nationally representative prevalence estimates and standard errors. Statistical tests of difference in the estimates between two time periods are based on a logistic regression model that accounts for the complex sampling design. Using a multinomial logit model, the time trend in tobacco use status is identified to capture the effects of macro level determinants including changes in tobacco control policies.

**Results:**

Between 2009 and 2012, overall tobacco use went down from 42.4% to 36.3%. The decline is more pronounced with respect to smokeless tobacco use than smoking. The prevalence of exclusive cigarette smoking went up from 7.2% to 10.6%; exclusive bidi smoking remained stable at around 2%; while smoking both cigarette and bidi went down from 4.6% to 1.8%; exclusive smokeless tobacco use went down from 20.2% to 16.9%; and both smokeless tobacco use and smoking went down from 8.4% to 5.1%. In general, the prevalence of tobacco use is higher among men, increases from younger to older age groups, and is higher among poorer people. Smoking prevalence is the highest among the slum population, followed by the tribal population, the national population and the border area population, suggesting greater burden of tobacco use among the disadvantaged groups.

**Conclusions:**

The overall decline in tobacco use can be viewed as a structural shift in the tobacco market in Bangladesh from low value products such as bidi and smokeless tobacco to high value cigarettes, which is expected with the growth in income and purchasing power of the general population. Despite the reduction in overall tobacco use, the male smoking prevalence in Bangladesh is still high at 37%. The world average of daily smoking among men is 31.1%. The Tobacco Control Act 2005 and the Amendment have yet to make a significant impact in curbing tobacco usage in Bangladesh. The findings in this paper further suggest that the tobacco control policies in Bangladesh need to include targeted interventions to restrain the use of particular types of tobacco products among specific demographic and socio-economic groups of the population, such as smoked tobacco among men, smokeless tobacco among women, and both smoked and smokeless tobacco among those living in rural areas, those in low socio-economic status and those belonging to the tribal and the slum population.

## Introduction

Smoking and passive smoking are collectively the biggest preventable cause of death in Bangladesh, with major public health burden of morbidity, disability, mortality and community costs [1,2]. An earlier epidemiological study conducted in 2004 showed that smoking was responsible for approximately 57 000 deaths and 1.2 million tobacco related illnesses per year in Bangladesh; 16% of all deaths among those of age 30 years and older were attributed to tobacco use [2]. A more recent study conducted using 2010 data concluded that about 25% of all deaths among men aged 25 to 69 years are attributable to smoking leading to average loss of 7 years of life per smoker [3]. These studies suggest an increase in the proportion of tobacco-attributable deaths. It alerts to the rapidly growing health and economic burden of tobacco use in Bangladesh and underscores the significance of controlling tobacco use.

Bangladesh is one of the top ten countries in the world with high current smoking prevalence of 44.7% among men [1]. This country is distinguished as the first signatory of the World Health Organization (WHO) Framework Convention on Tobacco Control (WHO FCTC) which was ratified on 10 May 2004. The ratification was made concrete with the passage of the Tobacco Control Act (TCA) on 15 March 2005. These tobacco control measures are expected to reduce smoking, or at the very least, to arrest the potential rise in smoking prevalence that has been observed in certain population groups [4].

The available studies of tobacco use in Bangladesh do not necessarily employ nationally representative samples. For example, Flora et al. (2009) estimated the prevalence of tobacco use in Bangladesh by gender and area using cross-sectional data collected from one urban area and one rural area during 2001, 2002, and 2003 [5]. According to this study, the overall prevalences of smoking, chewing and gul usage were 20.5%, 20.6% and 1.8% respectively. Current smoking and gul usage were significantly higher in males (42.2% and 2.2%, respectively) than females (2.3% and 1.5%, respectively) while chewing tobacco was more common in females (21.6%) than males (19.4%). Khan et al (2009) compared smoking prevalence of urban adult men between slum and non-slum areas in 2006 [6]. This study observed that male smoking prevalence for the total sample was 53.6% with significantly higher prevalence among men in slums (59.8%) than non-slums (46.4%). Sreeramreddy et al (2014), however, used a nationally representative sample from the Demographic and Health Survey 2007 to estimate the prevalence of smoking and smokeless tobacco use in nine south and south-east Asian countries including Bangladesh, although the study includes only male respondents for Bangladesh [7]. According to this study, male smoking prevalence was 60.01% and male smokeless tobacco use prevalence was 21.35%. In addition, for the purpose of surveillance, WHO conducted two nation-wide surveys—the Global Adult Tobacco Survey (GATS) in 2009 for the age group 15 years and above [1] and the Non-communicable Diseases (NCD) Risk Factor Survey 2010 for the age group 25 years and older [8]. The GATS found that 23.0% of adults aged 15 years or above were smokers of tobacco in Bangladesh (for males 44.7% and for females 1.5%). The NCD Risk Factor Survey observed 26.2% overall smoking prevalence—54.8% for men and 1.3% for women. The GATS and NCD Risk Factor Survey estimates are, however, not comparable due to the coverage of different age groups. In summary, the trend in tobacco use prevalence in Bangladesh cannot be determined with available estimates.

This paper examines the prevalence and patterns of tobacco use among adults in Bangladesh and the changes over time using large nationally representative, comparable and more recent surveys. The findings of the study will help policy makers in tracking the voluntary target for tobacco control policies in Bangladesh to achieve 30% relative reduction in tobacco use by 2025 as set by the World Health Assembly in 2013.

## Methods

This paper presents national level estimates of tobacco use prevalence in 2009 and 2012 based on household enumeration data (S1 File) collected as part of the International Tobacco Control (ITC) Bangladesh wave 1 and wave 3 surveys, conducted during February to May 2009 and November 2011 to May 2012, respectively. Both enumerations were done following exactly the same procedures. No enumeration was conducted in wave 2. In 2009, data on individual tobacco use, demographic characteristics, and household socio-economic status were collected on 95,454 adults aged 15 years and older from 31,688 households in 86 sampling areas. In 2012, these data were collected on 97,115 adult individuals from 33,295 households from the same sampling areas except for two slum areas that were abolished before the 2012 enumeration.

### Sampling Design

Four different sampling areas were selected for the study: (i) national sample, representative of the mainstream population, (ii) Garo and Chakma tribal sample, representative of the indigenous population, (iii) slum areas to represent the floating population, and (iv) border area sample to capture effects of cross-border trade of tobacco products on the population.

For the national sample, from six administrative divisions, 20 districts out of 64 were selected with probability proportional to population size. The number of districts from each division was determined on the basis of the distribution of the total population of the country across divisions. From the 20 districts, 37 upazilas/thanas (smallest administrative units of the local government) were selected with probability proportional to their respective population size. From each district, at least one upazila/thana was selected. From relatively larger districts in terms of population size, we selected two upazilas/thanas. From each upazila/thana, two villages/wards with probability proportional to population size were selected. Thus the total number of sample areas (villages/wards) from the mainstream population was 74.

While the national sample is a probability sample, the other three samples are purposive. First, four villages were selected from two districts (Netrokona and Rangamati) to survey the tribal communities of Garo and Chakma respectively. Second, two villages were selected from one district (Satkhira) to cover one land port that is used for cross-border trade between India and Bangladesh. Third, six slum areas from Dhaka city were selected in 2009 to survey tobacco use in the floating population. By 2012, two slum areas were demolished, thus leading to enumeration of four slum areas.

From the enumerated population in wave 1, approximately 6,000 respondents were randomly selected for individual in-depth interview through cohort surveys in waves 1, 2, and 3. The details of the randomization process are available in the ITC Project Technical Reports [9,10]. The purpose of the in-depth interview of sample respondents was to collect data on psychosocial and behavioural variables (e.g., beliefs and attitudes, perceived risk, subjective norms, perceived behavioural control, intentions to quit) that are influenced by tobacco control policies. These data are used to evaluate the effectiveness of tobacco control policies that affect tobacco use behaviours through a causal chain of psychosocial events and translate into economic and public health impacts.

The present paper is, however, based on the data collected in the wave 1 and wave 3 enumerations only. The data from the in-depth interviews was not directly used in the present analysis.

### Enumeration and Survey Procedures

From each village/ward, a maximum of 450 households were enumerated beginning from a randomly chosen end of the selected area. Only individuals living in private residences who were in their usual place of residence were enumerated. Businesses and institutions were excluded from the enumeration. The enumerators were allowed up to four visits to the same dwelling unit to collect complete household information. They scheduled revisits according to the convenience and availability of the informant from each household. Thus the enumerators were able to avoid non-response in all the sample areas except for one area in Dhaka city where they were denied access to apartment buildings that are strictly protected from unknown visitors.

From the head of each household, information was collected on the status of smoked and smokeless tobacco use of each adult member aged 15 years and older, housing conditions and demographics (gender and age). If the head was absent at the time of the enumeration, another household member identified as the key informant provided the necessary information. Based on the housing condition information such as structural condition of the house, area of the house, number of rooms, main materials of roof, floor and wall, source of drinking water, toilet facilities, fuel used for cooking, and possession and use of television and radio, a housing index was constructed that served as the basis for stratification of the population in each survey area by socio-economic status. The enumeration file thus allows us to determine the tobacco use status of individuals by region, gender, age, socio-economic status, and type of tobacco product (e.g., cigarette, bidi, and smokeless tobacco).

### Definition

An individual was identified as a smoker if he/she smokes cigarette or bidi or a combination of these products, at least once a week. If he/she did not meet this criterion, he/she was classified as a non-smoker. A similar principle was followed for the identification of smokeless tobacco users. Weekly use was used as a criterion to separate tobacco users from nonusers for conducting separate surveys for the two groups. The idea was to include occasional users in the user group who have some minimum frequency (e.g. weekly) in tobacco use. This definition of tobacco use on the basis of weekly use is broader than that based on daily use as it covers both regular and occasional users and may result in larger estimates of prevalence. We used in-depth survey data that collected information on daily use of tobacco and verified how closely the weekly tobacco use rate approximates the daily tobacco use rate. It turns out that out of all smokers who reported weekly use, 98.3% in wave 1 and 97.8% in wave 3 were daily smokers. However, this definition was followed consistently in the two waves of the enumeration and the estimates across the two waves are therefore comparable. Given the fact that occasional users are at high risk of turning into regular users, these estimates of prevalence reflect the extent of risky behaviour in the population as far as tobacco use is concerned.

### Descriptive analysis

From wave 1 and wave 3 enumeration, we obtained prevalence estimates for all tobacco products together and for smoking and smokeless tobacco use separately by gender, urban and rural areas, and socio-economic status of households for the national sample using logistic regression model. Parallel to the national sample, we obtained prevalence estimates for the tribal, slum and border samples. The purpose of comparing national level estimates with the estimates obtained from the purposive samples is to envisage how tobacco consumption pattern may differ among population sub-groups and minorities from the mainstream population. The prevalence estimates were adjusted for age, gender and socio-economic status of individual respondents. For each enumerated household in the mainstream population, household weights were calculated to account for the sampling design. These weights were used to obtain a nationally representative estimate of the prevalence of tobacco use. Associated standard errors and 95% confidence intervals (CIs) were constructed taking into account the sampling weights and complex survey methods. Chi-square tests were done based on logistic regression model to determine whether the differences in the prevalence estimates between the two years are statistically significant.

### Regression analysis

In order to explain the differences in prevalence estimates across sub-populations and over time, the status of tobacco use was classified into non-use of tobacco products, exclusive cigarette use, exclusive bidi use, dual use of cigarette and bidi, exclusive use of smokeless tobacco, and mixed use of smoked and smokeless tobacco. Both the national sample and the purposive samples (tribal, slum and border) were included in the analysis. Using a multinomial logit model, the tobacco use status variable was regressed on individual demographic characteristics such as gender and age, household socio-economic status, area of residence (urban/rural), type of sample (national/tribal/slum/border), fixed effects for upazillas/thanas, and finally the time trend capturing the effects of changes in the macro level determinants, such as economic development, growth and tobacco control policy environment. The time effect in the model reflects the impact of changes in the policy variables that affect the dependent variable at the aggregate level. The marginal effect of the time trend variable was estimated to identify the sign and magnitude of the change in the prevalence of a particular type of tobacco use status happening between 2009 and 2012 after controlling for observed socio-economic and locational characteristics. The standard errors of estimated coefficients account for the complex sampling design, in particular the household clusters at the village level.

### Ethics statement

The International Tobacco Control (ITC) Bangladesh Survey was conducted by the Bureau of Economic Research at the University of Dhaka, in collaboration with the International Tobacco Control (ITC) Bangladesh Project team, at the University of Waterloo, Canada. The survey protocol was approved by the Ethical Review Committees of the Bangladesh Medical Research Council and the University of Waterloo. Free and informed written consent of the subjects was obtained prior to the interview.

## Results

### Sample description

The sample descriptives including the percentage of males, urban areas and the age distribution of the enumerated population are presented by the type of sample in Table 1 below. The national sample reflects the national level proportion of urban population. Among the purposive samples, the tribal and border samples are all rural, while the slum sample is taken from the urban areas only in and around the capital city Dhaka. The gender and age distributions of the purposive samples closely reflect those of the national sample.

**Table 1:** Description of the study population by sample type, International Tobacco Control (ITC) Study Bangladesh Enumeration.

	National sample		Tribal sample		Slum sample		Border sample		Total	
	2009	2012	2009	2012	2009	2012	2009	2012	2009	2012
Number of sampling areas (villages/wards)	74	74	4	4	6	4	2	2	86	84
Number of households	29 899	31 452	788	892	552	501	449	450	31 688	33 295
Number of individuals	89 628	91 613	2 782	2 637	1 637	1 520	1 407	1 345	95 454	97 115
% of male	50.63	51.76	52.34	50.06	52.78	52.91	51.88	51.23	50.73	51.73
% of urban	33.53	28.14	0	0	100	100	0	0	33.20	28.11
Age distribution (%)										
15–17	8.43	7.80	6.37	4.97	8.98	8.18	8.10	8.03	8.38	7.73
18–24	20.16	18.94	18.38	15.62	27.31	21.74	20.11	19.70	20.23	18.91
25–39	35.43	35.83	36.04	35.65	35.55	34.24	33.05	34.13	35.42	35.77
40–54	22.71	23.39	22.16	27.72	19.91	23.01	23.45	22.75	22.66	23.50
55+	13.27	14.04	17.05	16.04	8.28	12.83	15.28	15.39	13.32	14.10

Source: ITC Bangladesh Enumeration, 2009 and 2012 (S1 File).

### Comparison of wave 1 and wave 3 estimates of the national sample

Between 2009 and 2012, the overall tobacco use prevalence went down in Bangladesh (Table 2). Between 2009 and 2012, the use of any type of tobacco decreased from 42.4% to 36.3%, which is explained by reduction in smokeless tobacco use from 28.6% to 22% to a large extent. This decline in prevalence resulted in about 4 million fewer tobacco users.

**Table 2:** Crude prevalence of tobacco use and number of tobacco users in Bangladesh, 2009–2012.

	**Wave 1: 2009**				**Wave 3: 2012**					
**Prevalence**										
	**%**	**SE**	**(95%**	**CI)**	**%**	**SE**	**(95%**	**CI)**	**Chi-square**	**p-value**
Any type of tobacco	42.4	1.35	(39.6,	45.1)	36.3	1.49	(33.5,	39.4)	25.15	< .001
Cigarette and/or bidi smoking	22.2	0.58	(21.1,	23.3)	19.4	0.98	(17.6,	21.4)	12.68	< .001
Smokeless tobacco	28.6	1.83	(25.1,	32.3)	22.0	1.46	(19.2,	24.9)	34.47	< .001
**Number of tobacco users (million)**			**Wave 1: 2009**		** **	**Wave 3: 2012**			**Change**	
Any type of tobacco			42.9				39.0		-3.9	
Cigarette and/or bidi			22.5				20.9		-1.6	
Smokeless tobacco			28.9				23.6		-5.4	

Source: ITC Bangladesh Enumeration, 2009 and 2012 (S1 File); Population data from the World Development Indicators Database.

Notes

1. Point estimates, standard errors and confidence intervals are obtained from logistic regression models accounting for complex sampling design estimated using SAS.

2. The number of cigarette and/or bidi smokers and smokeless tobacco users do not add up to the total number of users of tobacco because of the existence of mixed use of smoked and smokeless tobacco products by some persons.

### Prevalence of tobacco use by gender

The prevalence of tobacco use is generally higher among men than women (Table 3). The rate of smokeless tobacco use, on the other hand, is higher among women than men. The smoking prevalence fell from 22.2% to 19.4%, which is primarily driven by the decrease in the male smoking prevalence from 42% to 37%. The female smoking prevalence also decreased slightly from 1.8% to 0.9%. The male smoking prevalence, however, remained high. The global average of daily smoking among men is 31.1% [11]. The estimate from the present study is based on weekly use and hence can slightly overestimate the daily use rate. Both men and women experienced significant reduction in smokeless tobacco use during this period.

**Table 3:** Crude adult prevalence of tobacco use in Bangladesh by gender, 2009–2012.

	Wave 1: 2009				Wave 3: 2012				Chi-	
Tobacco Use	%	SE	(95%	CI)	%	SE	(95%	CI)	square	p-value
**Any type of tobacco**										
Male	53.2	1.25	(50.8,	55.7)	47.1	1.78	(43.6,	50.6)	13.76	< .001
Female	31.2	1.56	(28.2,	34.3)	24.9	1.45	(22.2,	27.9)	53.67	< .001
**Smoked tobacco**										
Male	42.0	0.90	(40.3,	43.8)	37.0	1.72	(33.7,	40.4)	12.41	< .001
Female	1.8	0.52	(1.0,	3.1)	0.9	0.43	(0.3,	2.3)	5.96	.015
**Smokeless tobacco**										
Male	26.8	2.16	(22.8,	31.2)	19.5	1.72	(16.4,	23.1)	25.64	< .001
Female	30.4	1.63	(27.3,	33.7)	24.5	1.42	(21.9,	27.4)	43.98	< .001

Source: ITC Bangladesh Enumeration, 2009 and 2012 (S1 File).

Note: Point estimates, standard errors and confidence intervals are obtained from logistic regression models accounting for complex sampling design estimated using SAS. Chi-square and p-values refer to the difference in the point estimates between wave 1 and wave 3.

### Prevalence of tobacco use by area of residence

The prevalence of tobacco use is greater among rural residents than the urban residents (Table 4). The gradient is larger for smokeless tobacco use than for smoking. Between 2009 and 2012, tobacco use decreased in both rural and urban areas with respect to all kinds of tobacco products.

**Table 4:** Crude adult prevalence of tobacco use in Bangladesh by area of residence, 2009–2012.

	Wave 1: 2009				Wave 3: 2012				Chi-	
Tobacco Use	%	SE	(95%	CI)	%	SE	(95%	CI)	square	p-value
**Any type of tobacco**										
Urban	36.2	1.49	(33.4,	39.3)	30.9	1.66	(27.7,	34.3)	13.15	< .001
Rural	45.1	1.28	(42.6,	47.6)	38.5	1.29	(36.0,	41.0)	38.77	< .001
**Smoked tobacco**										
Urban	19.9	0.70	(18.5,	21.3)	16.2	1.07	(14.2,	18.4)	10.98	< .001
Rural	23.2	0.69	(21.9,	24.6)	20.7	0.96	(18.9,	22.6)	10.04	.002
**Smokeless tobacco**										
Urban	21.8	2.22	(17.8,	26.5)	18.1	1.38	(15.6,	21.0)	6.25	.025
Rural	31.5	1.62	(28.4,	34.8)	23.5	1.64	(20.4,	26.8)	37.74	< .001

Source: ITC Bangladesh Enumeration, 2009 and 2012 (S1 File).

Note: Point estimates, standard errors and confidence intervals are obtained from logistic regression models accounting for complex sampling design estimated using SAS. Chi-square and p-values refer to the difference in the point estimates between wave 1 and wave 3.

### Prevalence of tobacco use by age group

The prevalence of tobacco use increases consistently from younger to older age groups (Table 5). In 2009, overall tobacco prevalence was the highest (70.9%) among the oldest 55+ age group, with 28.9% smoking prevalence and 56.2% smokeless tobacco prevalence. With respect to smoking prevalence, however, the highest rate (32.2%) was observed among 40–54 age group. In 2012, the patterns of tobacco use by age group remained the same. However, every age group experienced reduction in prevalence with respect to every kind of tobacco product except age group 55+ for smoked tobacco and age group 15–17 for smokeless tobacco. In particular, for age group 40–54, prevalence of any type of tobacco use fell by 10 percentage points from 64% to 54%, which is again largely attributable to reduction in smokeless tobacco use by 11 percentage points.

**Table 5:** Crude adult prevalence of tobacco use in Bangladesh by age group, 2009–2012.

	Wave 1: 2009				Wave 3: 2012				Chi-	
Tobacco Use	%	SE	(95%	CI)	%	SE	(95%	CI)	square	p-value
**Any type of tobacco**										
15–17	4.6	0.57	(3.6,	5.8)	2.8	0.51	(2.0,	4.0)	7.06	.008
18–24	16.3	1.23	(14.1,	18.9)	12.0	1.08	(10.1,	14.3)	20.44	< .001
25–39	41.5	1.80	(38.1,	45.1)	33.3	1.91	(29.7,	37.2)	37.31	< .001
40–54	64.0	1.76	(60.5,	67.4)	54.2	2.22	(49.8,	58.5)	45.47	< .001
55+	70.9	1.97	(66.8,	74.6)	64.2	2.66	(58.9,	69.2)	17.74	< .001
**Smoked tobacco**										
15–17	3.3	0.32	(2.7,	4.0)	1.8	0.23	(1.4,	2.3)	20.94	< .001
18–24	10.6	0.48	(9.8,	11.6)	8.1	0.46	(7.2,	9.0)	20.27	< .001
25–39	24.3	0.65	(23.0,	25.6)	20.6	1.10	(18.5,	22.8)	18.07	< .001
40–54	32.2	1.10	(30.1,	34.4)	27.9	1.37	(25.3,	30.7)	25.89	< .001
55+	28.9	1.69	(25.7,	32.4)	26.9	2.09	(23.0,	31.1)	1.94	.164
**Smokeless tobacco**										
15–17	1.7	0.52	(0.9,	3.1)	1.2	0.50	(0.5,	2.7)	0.95	.329
18–24	7.9	1.41	(5.6,	11.2)	4.9	1.13	(3.1,	7.7)	16.92	< .001
25–39	25.1	2.33	(20.8,	30.0)	16.9	2.06	(13.2,	21.3)	34.16	< .001
40–54	45.8	2.58	(40.8,	50.9)	34.8	2.11	(30.8,	39.0)	43.14	< .001
55+	56.2	2.24	(51.8,	60.5)	47.1	2.19	(42.8,	51.4)	38.67	< .001

Source: ITC Bangladesh Enumeration, 2009 and 2012 (S1 File).

Note: Point estimates, standard errors and confidence intervals are obtained from logistic regression models accounting for complex sampling design estimated using SAS. Chi-square and p-values refer to the difference in the point estimates between wave 1 and wave 3.

### Prevalence of tobacco use by socio-economic status

The prevalence of tobacco use—both smoked and smokeless tobacco use—was higher among poorer people in Bangladesh in both 2009 and 2012 (Table 6). Compared to smoking, the gradient is greater for smokeless tobacco use. Between 2009 and 2012, people across all socio-economic status experienced decrease in tobacco use of all types.

**Table 6:** Crude adult prevalence of tobacco use in Bangladesh by socio-economic status, 2009–2012.

	WAVE 1: 2009				WAVE 3: 2012				Chi-	
Tobacco Use	%	SE	(95%	CI)	%	SE	(95%	CI)	square	p-value
**Any type of tobacco**										
Low	49.6	1.46	(46.7,	52.4)	43.0	1.30	(40.5,	45.6)	33.16	< .001
Middle	43.0	1.43	(40.2,	45.8)	36.8	1.87	(33.2,	40.5)	16.66	< .001
High	36.4	1.47	(33.6,	39.3)	30.6	1.54	(27.6,	33.7)	20.97	< .001
**Smoked tobacco**										
Low	27.4	0.78	(25.9,	29.0)	24.0	0.95	(22.2,	25.9)	19.73	< .001
Middle	22.4	0.63	(21.2,	23.6)	19.5	1.27	(17.1,	22.1)	7.72	.005
High	18.0	0.68	(16.7,	19.4)	15.7	0.90	(14.0,	17.6)	9.09	.003
**Smokeless tobacco**										
Low	33.2	2.13	(29.2,	37.5)	25.4	1.56	(22.4,	28.6)	34.51	< .001
Middle	29.2	1.99	(25.4,	33.2)	22.6	1.58	(19.7,	25.9)	28.90	< .001
High	24.5	1.66	(21.4,	27.9)	18.6	1.46	(15.9,	21.6)	29.28	< .001

Source: ITC Bangladesh Enumeration, 2009 and 2012 (S1 File).

Note: Point estimates, standard errors and confidence intervals are obtained from logistic regression models accounting for complex sampling design estimated using SAS. Chi-square and p-values refer to the difference in the point estimates between wave 1 and wave 3.

### Prevalence of tobacco use by sample type

Comparison across sample types shows that smoking prevalence (adjusted for age, gender and socio-economic status) is the highest among the slum sample, followed by the tribal sample, the national sample and the border area sample (Table 7). This pattern did not change from 2009 to 2012, although the percentage decreased invariably across all samples between the two years. The breakdown of smoking prevalence by gender reveals that although smoking is rare among women in the general population, it is significantly high among tribal women.

**Table 7:** Adjusted prevalence of tobacco use in Bangladesh by gender across sample types, 2009–2012.

**Wave 1**		**National**				**Border**				**Tribal**				**Slum**			
**Tobacco Use**	**Sex**	**%**	**SE**	**(95%**	**CI)**	**%**	**SE**	**(95%**	**CI)**	**%**	**SE**	**(95%**	**CI)**	**%**	**SE**	**(95%**	**CI)**
Any type of tobacco	Male	53.0	1.20	(50.7,	55.4)	53.3	1.88	(49.6,	57.0)	49.3	1.93	(45.6,	53.1)	80.6	1.77	(76.9,	83.9)
	Female	32.8	1.60	(29.8,	36.0)	32.4	1.65	(29.2,	35.7)	26.5	1.55	(23.6,	29.6)	36.6	2.81	(31.3,	42.2)
	All	43.2	1.34	(40.6,	45.8)	43.1	1.41	(40.4,	45.9)	38.2	1.48	(35.3,	41.2)	60.0	1.44	(57.1,	62.8)
Smoked tobacco	Male	42.1	0.90	(40.4,	43.9)	37.3	2.10	(33.3,	41.5)	47.8	1.89	(44.1,	51.5)	78.3	1.92	(74.3,	81.9)
	Female	1.8	0.53	(1.0,	3.2)	0.1	0.15	(0.0,	1.1)	16.5	1.27	(14.2,	19.2)	1.3	0.52	(0.6,	2.8)
	All	22.7	0.59	(21.5,	23.8)	19.4	1.10	(17.3,	21.6)	32.9	1.27	(30.5,	35.4)	41.4	0.98	(39.5,	43.4)
Smokeless tobacco	Male	26.4	2.08	(22.5,	30.7)	34.3	1.65	(31.1,	37.6)	12.0	1.48	(9.4,	15.2)	19.1	2.20	(15.2,	23.8)
	Female	32.1	1.66	(29.0,	35.5)	32.4	1.63	(29.3,	35.6)	13.6	1.32	(11.2,	16.4)	36.7	2.90	(31.2,	42.6)
	All	29.1	1.80	(25.7,	32.8)	33.4	1.35	(30.8,	36.1)	12.6	1.19	(10.5,	15.2)	27.3	2.19	(23.2,	31.8)
**Wave 3**		**National**				**Border**				**Tribal**				**Slum**			
**Tobacco Use**	**Sex**	**%**	**SE**	**(95%**	**CI)**	**%**	**SE**	**(95%**	**CI)**	**%**	**SE**	**(95%**	**CI)**	**%**	**SE**	**(95%**	**CI)**
Any type of tobacco	Male	45.6	1.73	(42.2,	49.0)	42.5	2.16	(38.3,	46.8)	47.4	1.85	(43.8,	51.0)	68.4	4.12	(59.9,	75.9)
	Female	25.5	1.47	(22.7,	28.5)	21.0	1.62	(18.0,	24.3)	23.7	1.36	(21.1,	26.4)	25.7	3.58	(19.3,	33.3)
	All	35.9	1.41	(33.2,	38.7)	32.1	1.46	(29.3,	35.1)	35.8	1.26	(33.4,	38.3)	47.8	3.10	(41.8,	53.9)
Smoked tobacco	Male	36.3	1.61	(33.2,	39.5)	31.0	2.25	(26.8,	35.6)	45.0	1.74	(41.6,	48.4)	62.0	5.09	(51.6,	71.4)
	Female	0.9	0.43	(0.3,	2.3)	0.3	0.22	(0.1,	1.2)	9.9	0.93	(8.2,	11.9)	0.1	0.06	(0.0,	0.3)
	All	19.2	0.91	(17.4,	21.0)	16.2	1.16	(14.1,	18.6)	28.1	1.09	(26.1,	30.3)	32.0	2.54	(27.3,	37.2)
Smokeless tobacco	Male	18.5	1.63	(15.5,	21.9)	16.9	1.45	(14.2,	19.9)	8.8	1.01	(7.0,	11.0)	17.2	3.32	(11.6,	24.7)
	Female	25.2	1.41	(22.6,	28.1)	20.8	1.65	(17.7,	24.2)	16.1	1.26	(13.7,	18.7)	25.8	3.56	(19.4,	33.4)
	All	21.7	1.43	(19.0,	24.6)	18.7	1.26	(16.4,	21.3)	12.3	0.93	(10.6,	14.2)	21.2	1.80	(17.9,	25.0)

Source: ITC Bangladesh Enumeration, 2009 and 2012 (S1 File).

Note: Prevalence estimates are adjusted for age group, gender and socio-economic status and control for the complex sampling design. Point estimates, standard errors and confidence intervals are obtained from logistic regression models accounting for complex sampling design estimated using SAS -callable SUDAAN.

While smoking prevalence was the lowest in the border sample, smokeless tobacco use prevalence was the highest in this sample in 2009, followed by the national, slum, and tribal samples in that order (Table 7). By 2012, the order of samples, however, changed because of a dramatic decline in smokeless tobacco use in the border sample from 34.3% to 16.9% among men, from 32.4% to 20.8% among women and from 33.4% to 18.7% overall. In 2012, the national prevalence of smokeless tobacco use became the highest followed by the slum, border and tribal samples.

Smokeless tobacco use appears to be the lowest among the tribal people in both years. However, the prevalence among females increased from 15.1% to 17.3% between 2009 and 2012 indicating potential growth of smokeless tobacco use in the indigenous population.

### Prevalence of tobacco use by type and mix of tobacco products

The breakdown of tobacco users by type and mix of tobacco products and comparison between 2009 and 2012 reveal a number of remarkable shifts happening in the tobacco market in Bangladesh (Table 8):

1. The prevalence of exclusive cigarette smoking went up from 7.2% to 10.6%.
2. The prevalence of exclusive bidi smoking remained stable at around 2%.
3. The prevalence of smoking both cigarette and bidi went down from 4.6% to 1.8%.
4. The prevalence of exclusive smokeless tobacco use went down from 20.2% to 16.9%.
5. The prevalence of both smokeless and smoked tobacco use went down from 8.4% to 5.1%.

**Table 8:** Crude adult prevalence of tobacco use in Bangladesh by type and mix of tobacco products, 2009–2012.

** **	**Wave 1: 2009**			**Wave 3: 2012**			**Chi-**	
**Tobacco Use**	**%**	**SE**	**(95% CI)**	**%**	**SE**	**(95% CI)**	**Square**	**p-value**
Exclusively smokes cigarettes	7.2	0.50	(6.2, 8.2)	10.6	0.65	(9.4, 11.9)	36.26	< .001
Exclusively smokes bidi	2.0	0.32	(1.5, 2.7)	1.9	0.30	(1.4, 2.6)	0.20	.653
Smokes cigarettes AND bidi	4.6	0.43	(3.9, 5.6)	1.8	0.25	(1.4, 2.4)	64.66	< .001
Uses only smokeless tobacco (does NOT smoke)	20.2	1.21	(17.9, 22.7)	16.9	0.96	(15.1, 18.9)	27.33	< .001
Smokes AND uses smokeless	8.4	0.75	(7.0, 10.0)	5.1	0.66	(3.9, 6.5)	25.61	< .001
**Number of tobacco users (million)**	**Wave 1: 2009**				**Wave 3: 2012**		**Change**	
Exclusively cigarette smoker		7.3			11.4		4.2	
Exclusively bidi smoker		2.0			2.1		0.0	
Dual smoker of cigarette and bidi		4.7			2.0		-2.7	
Exclusively smokeless tobacco user		20.5			18.1		-2.3	
Both smoker and smokeless tobacco user		8.5			5.5		-3.0	
Total		42.9			39.0		-3.9	

Source: ITC Bangladesh Enumeration, 2009 and 2012 (S1 File); Population data from the World Development Indicators Database.

Note: Point estimates, standard errors and confidence intervals are obtained from logistic regression models accounting for complex sampling design estimated using SAS. Chi-square and p-values refer to the difference in the point estimates between wave 1 and wave 3.

In terms of the number of tobacco users, it appears that the market for exclusive cigarette use expanded significantly with 4.15 million additional smokers in three years’ time, that is, about 1.4 million smokers a year (Table 8). At the average consumption of 9.3 cigarettes per day per smoker (ITC Wave 3 Survey), this increase in the number of cigarette smokers means additional consumption of 4.7 billion cigarettes a year.

On the other hand, the number of dual smokers and mixed tobacco users went down. The average daily consumption for dual smokers is lower than that for the exclusive cigarettes smokers (5.7 cigarettes per day), while the average daily consumption of mixed smokers is equal (9.3 cigarettes per day). Thus, the loss of cigarette consumption from the reduction of 2.7 million dual smokers and 3 million mixed tobacco users is expected to be 5.34 billion pieces, which more than offsets the increase in cigarette consumption from the growth in exclusive cigarette use. In other words, the net cigarette consumption decreased over 2009–2012.

Note that the dual smokers and mixed tobacco users are the occasional cigarette smokers and are likely to be more sensitive to cigarette price increases. Between 2009 and 2012, both the retail prices of cigarettes and the supplementary duty on cigarettes were raised consistently every year. As a consequence, some of these *peripheral* cigarette smokers were likely the first to quit.

As the TCA was passed in 2005 and the tax and non-tax measures were implemented in the subsequent period, it can be conjectured that the significant decline in the overall tobacco use prevalence in Bangladesh has been brought forth by the nationwide tobacco control programs. It is likely that there was a strong time effect between 2009 and 2012 that drove the overall tobacco prevalence down in the country.

In order to identify this effect, we ran a multinomial logit regression of the status of tobacco use by type and mix of tobacco products. The estimates are provided in Table 9. The time effect is positive for exclusive cigarette use, not significant for exclusive bidi use, while negative for other types of exclusive or mixed use. It indicates that the overall change in the macro environment including the strengthening of the tobacco control policy measures and the structural decline in the bidi industry in the recent decade was able to create a negative impact on tobacco use prevalence except for the exclusive cigarette users. The estimated marginal time effects show that between 2009 and 2012, the prevalence of smoking exclusively cigarettes went up by 3 percentage points, the prevalence of smoking exclusively bidi did not change, while the prevalences of smoking both cigarette and bidi, of using exclusively smokeless tobacco and of mixed tobacco use went down by 2 to 4 percentage points. Overall the trend in the reduction in tobacco use is 6 percentage points given by the sum of the marginal effects for different tobacco products between 2009 and 2012. At this linear trend rate, other things remaining the same, Bangladesh is way over the target to achieve 30% relative reduction in the 2010 base year level of tobacco use by 2025 as set by the World Health Assembly in 2013. This result supports the finding of the WHO Global Report on Trends in Prevalence of Tobacco Smoking 2015 that shows that based on current smoking trends Bangladesh will achieve the smoking component of the target. WHO projects that if tobacco control efforts continue at the same intensity, around 15% of the population will be smokers in 2025 [12].

**Table 9:** Estimates of marginal effects on the probability of tobacco use from the multinomial logit regression of tobacco use status by type and mix of tobacco products in Bangladesh, 2009–2012.

	Exclusively cigarette		Exclusively bidi		Both cigarette and bidi		Exclusively smokeless		Both smoked and smokeless	
**Time effect**	**0.03**	***	**-0.00**		**-0.02**	***	**-0.03**	***	**-0.04**	***
Female	-0.19	***	-0.03	***	-0.05	***	0.18	***	-0.11	***
Age	0.00	***	0.00	***	0.00	***	0.01	***	0.00	***
Household socio-economic status										
Medium	-0.01	***	-0.01	***	-0.01	***	-0.02	***	-0.02	***
High	-0.01	***	-0.02	***	-0.02	***	-0.04	***	-0.04	***
Rural area of residence	-0.02		0.01	***	0.01	***	0.01	***	0.02	***
Type of sample										
Tribal sample	0.02	***	0.03	***	0.03	***	-0.21	***	-0.01	
Slum sample	0.06	***	0.03	**	0.00		0.08	***	0.05	***
Border area sample	0.01		-0.01	**	-0.01		0.04	***	0.03	***

Source: Estimated from ITC Bangladesh Enumeration, 2009 and 2012 (S1 File).

Notes

1. Pseudo R^2^ = 0.2671.

2. *** and ** indicate significant at 1% and 5% levels respectively.

3. The number of observations is 192,449.

4. The base categories include the male national sample in low socio-economic status, living in urban areas, in year 2009. The marginal effects on probability represent the change in probability from a discrete change from the base category.

5. The base outcome is no use of tobacco products.

6. The estimates of the fixed effects for the upazilas/thanas are suppressed.

7. The standard errors of estimated coefficients account for the complex sampling design, in particular the household clusters at the village level.

## Discussion

Although low- and middle-income countries (LMICs) generally lag behind high-income countries in tobacco control, Bangladesh is noted as one of those countries in the LMICs that have implemented tobacco control policies effectively since the early 1990s [13]. It was a very timely initiative on the part of the government of Bangladesh to implement the TCA in 2005 that banned smoking in public places and transports, advertising and promotion, and tobacco product vending machines. In addition, it required 30% text warning labels on the packets of smoked tobacco products. The TCA 2005 was followed by a series of tobacco tax measures, including upward adjustments in the price tiers of cigarettes with differential tax rates, raising cigarette tax rates, introducing taxes on smokeless tobacco products, and introducing duty on tobacco exports. The decline in tobacco use in Bangladesh is attributable to not only the policy changes occurring during the period under observation, but also to the cumulative effect of the tobacco control initiatives that took root in the country over a long past.

The study observed that the reduction in tobacco use in Bangladesh over the period under observation from 2009 to 2012 came disproportionately from the reduction in the use of smokeless tobacco. In 2008, for the first time, smokeless tobacco was brought under the tobacco control mechanism by imposing 15% value added tax on zarda (chewing tobacco) and gul (oral powder) which are the most common forms of smokeless tobacco products in the country. It was followed by the introduction of 10% supplementary duty on the ex-factory price of zarda and gul in 2009. The supplementary duty was further raised to 20% in 2010–11 and to 30% in 2011–12. The significant time effect present in both exclusive smokeless tobacco use and mixed use of smokeless and smoked tobacco indicates that the negative effect of the increase in tax that was presumably passed on to the price increase was at work in inducing smokeless tobacco users to quit. The negative effect of tax and price increase on smokeless tobacco use in Bangladesh has been confirmed in a separate paper based on the International Tobacco Control (ITC) study [14]. Besides, the remarkable decrease in smokeless tobacco use in the border sample as noted in the previous section requires further investigation to determine if this dramatic decrease has anything to do with the banning of smokeless tobacco products in India that came into effect in 2011 and consequent slowdown in the supply of smokeless tobacco products to the areas in Bangladesh close to the border with India.

On the other hand, the prevalence of exclusive cigarette use went up, although the use of cigarettes in conjunction with bidi or smokeless tobacco went down. This finding portends the potential for the expansion of cigarette industry in future in Bangladesh while the bidi and smokeless tobacco industry are gradually contracted. The overall decline in tobacco use prevalence can therefore be viewed as a structural shift in the tobacco market in Bangladesh from low value products such as bidi and smokeless tobacco to high value cigarettes, which is expected with the growth in income and purchasing power of the general population.

For those who switched to exclusive cigarette use, the increase in the affordability of cigarettes triggered the trading up. Based on the percentage of daily income spent on an average dose of cigarettes for smokers, the International Tobacco Control (ITC) Project (2012) report observed that cigarettes became 7.63% more affordable between 2009 and 2010 [15]. It happened despite the negative effect on cigarette demand of tax and government declared price increases that fell short of the positive effect of per capita income growth at 4–5% per annum [16].

The price and tax of bidi did not change over the period under observation. As a result, the real price of bidi went down which should have spurred the demand for bidi. To the contrary, the prevalence of exclusive bidi smokers did not change while that of dual smokers of bidi and cigarette and of mixed smokers using both smokeless tobacco and cigarette/bidi went down. It indicates a certain degree of complementarity between cigarette and bidi for dual users and between smoked and smokeless tobacco for mixed users. The increase in the price and tax on cigarettes had a negative impact not only on the decision to smoke cigarettes, but also on the choices of bidi and smokeless tobacco products for the multiple product users. Similarly, the increase in the tax and presumably the consequent increase in the price of smokeless tobacco also negatively influenced the decision to smoke cigarette and/or bidi for this group.

In addition, the pattern of tobacco use by population sub-groups reveals some concerns that are yet to be addressed through targeted interventions in the tobacco control policy framework.

First, the prevalence of tobacco use was found greater among poorer people, who are therefore susceptible to heavier disease burden. Thus economic inequality has a direct correspondence with health inequality mediated by tobacco use. It also implies that poorer people have greater economic burden in the form of larger tobacco expenditure in relation to their income and higher cost of health care expenditure and lost productivity owing to tobacco-induced illnesses [17].

Second, although the growth in exclusive cigarette use largely affected the male population in the past, the tobacco industry can target the female population in a desperate effort to expand the market and profitability of cigarette production. Also the smokeless tobacco use among tribal women went up during 2009–2012 indicating the potential of expanding the smokeless tobacco market in the indigenous population.

Third, the relatively high prevalence of tobacco use in the rural areas indicates the heavier disease burden caused by tobacco use in these parts of the country where the outreach of health care facilities is limited compared to urban areas. The rural tobacco users are therefore less likely to seek and access medical care in the event of illnesses and are more likely to go untreated and internalize the sufferings.

Finally, the relatively higher smoking prevalence in the tribal and slum sample compared to the national average again points to the heavier disease burden in these populations that are also economically more vulnerable and are lagging behind the mainstream population.

## Limitations of the Study

The present study has two limitations of the survey design. First, the use of tobacco, particularly smoking is not openly discussed due to social taboo in Bangladesh. It is not uncommon for many parents/heads of households in Bangladesh to be unaware of the smoking status of their children. Therefore, actual prevalence may be underestimated. Second, the prevalence estimate in ITC data is based on tobacco use at least once a week, while the standard reporting method may be current or daily tobacco use. It is likely that the ITC estimates will be higher than the daily use prevalence and lower than the current use prevalence. In order to identify the reporting bias caused by these limitations, we compared the 2009 ITC estimates of smoking and smokeless tobacco use prevalence of men and women with the 2009 estimates obtained from the Global Adult Tobacco Survey (GATS) [1]. According to the GATS, the male prevalence of current smoking was 44.7% and the prevalence of daily smoking was 40.7% compared to 42% obtained from the ITC study. Apparently, for male smoking prevalence, the weekly use estimate is slightly higher than the daily use estimate and lower than the current use estimate. The female smoking prevalence estimates are 1.5% and 1.3% for current and daily smoking according to GATS, while the ITC estimate is higher than both (1.8%). For smokeless tobacco use, the GATS estimate for male prevalence is 26.4% and the ITC estimate is 26.8%; the GATS estimate for female prevalence is 27.9% and the ITC estimate is higher at 30.4%. Thus, it appears that the ITC data based weekly use estimates are generally higher than the daily use estimates. However, it is expected that the bias is present in both waves of data collection conducted under the same study protocol. Hence, the conclusion about the downward trend in tobacco use in Bangladesh observed in the present analysis can be maintained.

## Conclusions

Between 2009 and 2012, the overall tobacco use prevalence went down in Bangladesh. The decline is more pronounced with respect to smokeless tobacco use than smoking. This decline in prevalence resulted in about 4 million fewer tobacco users. The reduction in tobacco use in Bangladesh during 2009–2012 came disproportionately from the reduction in the use of smokeless tobacco indicating that the negative effect of the increase in tax on smokeless tobacco was at work. Despite tax and price increases for cigarettes occurring several times by 2012, the prevalence of exclusive cigarette use went up. The overall decline in tobacco use can be viewed as a structural shift in the tobacco market in Bangladesh from low value products such as bidi and smokeless tobacco to high value cigarettes, which is expected with the growth in income and purchasing power of the general population.

Despite the reduction in overall tobacco use, the male smoking prevalence in Bangladesh (37%) is quite high. The world average of daily smoking among men is 31.1%. We conclude that the Tobacco Control Act of 2005 and the subsequent Amendment of 2015 have yet to make a significant impact in curbing tobacco usage in Bangladesh.

The findings in this paper further suggest that the tobacco control policies in Bangladesh need to include targeted interventions to restrain the use of particular type of tobacco products among specific demographic and socio-economic groups of the population, such as smoked tobacco among men, smokeless tobacco among women, and both smoked and smokeless tobacco among those living in rural areas, those in low socio-economic status and those belonging to the tribal and the slum population.

## Supporting Information

S1 File ITC Bangladesh Enumeration, 2009 and 2012. The ITC Bangladesh Enumeration data for national sample for wave 1 2009 is in file **itcbangladeshcensusprs1.xlsx**. The ITC Bangladesh Enumeration data for national sample for wave 3 2012 is in file **itcbangladeshcensusprs3.xlsx**. The ITC Bangladesh Enumeration data for tribal, floating and border samples for wave 1 2009 is in file **itcbangladeshcensustrsl1.xlsx**. The ITC Bangladesh Enumeration data for tribal, floating and border samples for wave 3 2012 is in file **itcbangladeshcensustrsl3.xlsx**.
(ZIP)
